# The Role of the Inframammary Fold (IMF) in Aesthetic and Reconstructive Surgery: A Critical Analysis and Surgical Solution

**DOI:** 10.1007/s00266-023-03729-w

**Published:** 2023-11-13

**Authors:** Donald A Hudson

**Affiliations:** UCT Private Academic Hospital, Rondebosch, Cape Town, South Africa

**Keywords:** Inframammary fold, Breast surgery, Breast reduction, Breast reconstruction

## Abstract

**Introduction:**

The inframammary fold (IMF) is a critical structure in breast aesthetics and is affected by various types of breast surgery. The ideal IMF has a semi-elliptical shape, which may become attenuated with age and descends in macromastia. The aim of this study was to analyse the IMF and retain/restore its shape with sutures.

**Methods:**

A retrospective study was conducted on breast surgeries performed over a four-year period (2019–2022). The morphometry of the IMF was evaluated preoperatively while the patients were standing. In cases where the IMF was symmetrical, sutures were used to reinforce it during surgery. When the loss of the semi-elliptical shape was clinically indicated, the IMF was mobilized, repositioned, and then sutured into place.

**Results:**

The study included 56 patients: 43 undergoing immediate breast reconstruction, and 13 undergoing bilateral breast reductions. In over two thirds of the patients, the lateral IMF was inferiorly displaced compared to the medial IMF.

**Conclusion:**

It is recommended to reinforce the IMF in all patients undergoing breast surgery. Where the IMF has an elliptical shape preoperatively, it is reinforced. Where IMF is inferiorly displaced, mobilization and superior advancement of the IMF, followed by suture reinforcement, are necessary. This approach results in a well-defined IMF with improved breast aesthetics.

**Level of Evidence IV:**

This journal requires that authors assign a level of evidence to each article. For a full description of these Evidence-Based Medicine ratings, please refer to the Table of Contents or the online Instructions to Authors www.springer.com/00266.

**Supplementary Information:**

The online version contains supplementary material available at 10.1007/s00266-023-03729-w.

## Introduction

The inframammary fold (IMF) is a critical structure defining the breast and plays a significant role in breast aesthetics [1–3]. The "aesthetic" IMF is elliptical in shape and may even resemble a half-circle [4]. It is a well-defined and distinct structure [1–4].

The IMF changes with age [5, 6] and in breast hypertrophy, for example. It becomes attenuated with age [5, 6] and descends in macromastia, especially in patients with a high BMI [5].

The aim of this study was to assess the morphometry of the IMF in patients having breast reduction or reconstruction for cancer over a 4 year period (2019–2022) and then to create a crisp, aesthetic IMF.

## Methods

Retrospective analysis of all patients undergoing either breast reduction or breast reconstruction during a 4 year period (2019–2022).

### Methods

The patient is marked standing. The IMF is marked from its medial to its lateral extent. Additionally, the breast meridian is marked, dividing the IMF into 2 segments: medial and lateral. The anterior axillary line, starting at the axilla and passing vertically down through the lateral IMF is marked. The shape of the IMF is then critically examined while the patient is standing.

**Fig. 1:**
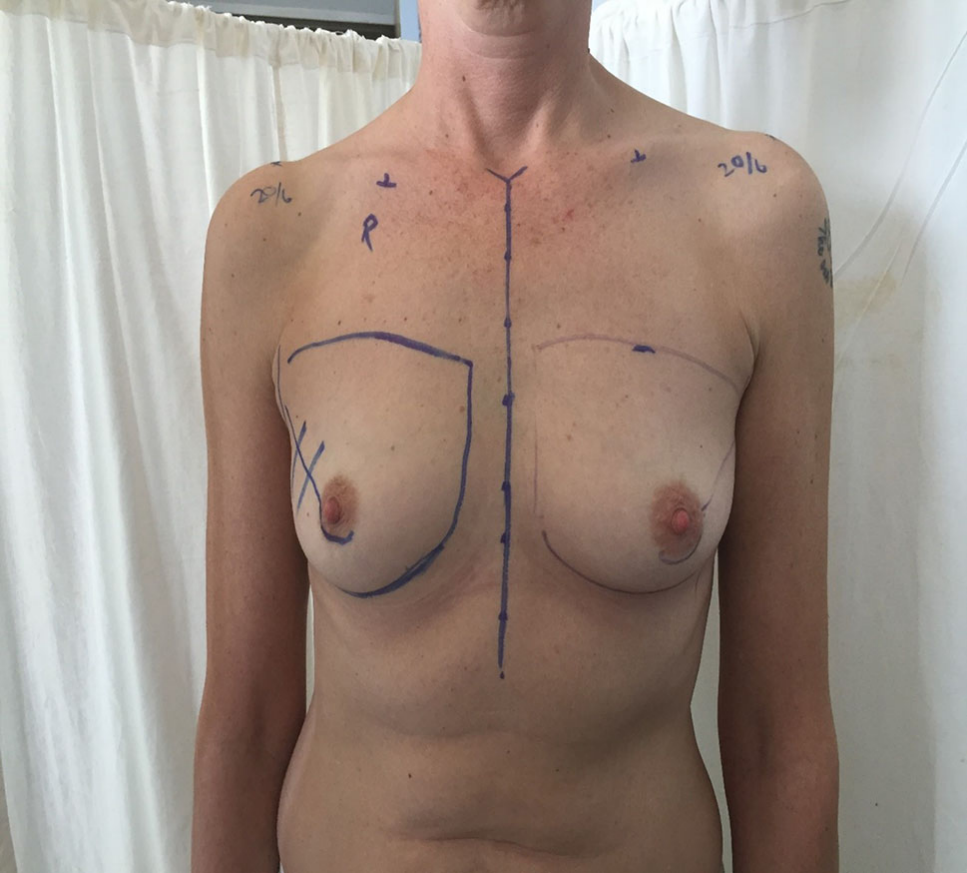
Patient for nipple sparing mastectomy and reconstruction. In this patient, the IMF has a semi-elliptical shape, and the lateral IMF is not displaced. This patient requires IMF suture reinforcement only prior to immediate reconstruction

Special attention should be paid to the shape of the lateral half of the IMF. Does the IMF form a symmetrical curve and end at the same horizontal level as the medial IMF (Fig. 1)? Or, is it "flat" in which case, the lateral extent of the IMF ends inferiorly to the medial IMF (Video 1)

When the lateral IMF is displaced inferiorly, this difference is measured. To do this, the position of the medial IMF is marked, and then a horizontal line is projected across the breast to its lateral aspect. A point is marked where this horizontal line intersects the vertical line of the anterior axillary fold. The difference is recorded in cm (see table 1).

**Table 1:** Note that it is the lateral half of the IMF which is displaced inferiorly

Breast types	Total	Normal IMF	Displaced at IMF
Breast reduction	13	3	10
Prosthetic reconstruction after mastectomy	43	13	30

It is important to note that the malposition or flattening of the IMF is still present in a supine position, but the inferior displacement of the lateral IMF is less noticeable in the supine position.

### Surgical Technique

There are 2 possibilities depending on IMF configuration; (i) reinforcing the IMF, or, (ii) mobilizing, superior repositioning of the lateral IMF, and then suture reinforcement of the IMF. In all cases, tumescent infiltration of local anaesthetic fluid is injected along the IMF.

#### Reinforcing the IMF

In this situation the shape/configuration of the iMF is adequate—forming a semi-ellipse.

The IMF is reinforced with a double armed 1/0 Maxon suture. The first suture is inserted at the level of the breast meridian. A second suture is inserted in the middle of the medial half of the IMF. Two or three sutures are inserted into the lateral half of the IMF, i.e. at the anterior axillary fold, in the middle of the lateral half of the IMF, and one suture is inserted between these two.

The first bite of the suture is horizontal and passes from the pectoral fascia through the rib periosteum and exits through the pectoral fascia. This is the anchoring part of the suture. Then, the suture passes through the superficial fascial system (SFS), after which the needle is tilted to pass horizontally in a subdermal plane, along the originally marked IMF. The suture then passes back through the SFS and is tied.

#### Repositioning the IMF: (Videos 2 and 3)

This technique is used when the IMF, especially the lateral IMF, is amorphous.

In this situation, the time honoured principle in plastic surgery is applied. Viz

i) Mobilize the lateral IMF—at the level of the pectoral and serratus fascia.
ii) Reposition the lateral IMF: A line is drawn on the chest wall/pectoral fascia marking the new lateral aspect of the IMF.
iii) Fixing the IMF: The IMF is fixed using a suture, as described above. The new position where the IMF is fixed and reinforced is made intraoperatively. The surgeon uses clinical judgement and experience as to where the IMF should be repositioned in order to recreate the semi-elliptical shape of the IMF. The lateral end of the IMF should be at the same level as the medial IMF

**Fig. 2:**
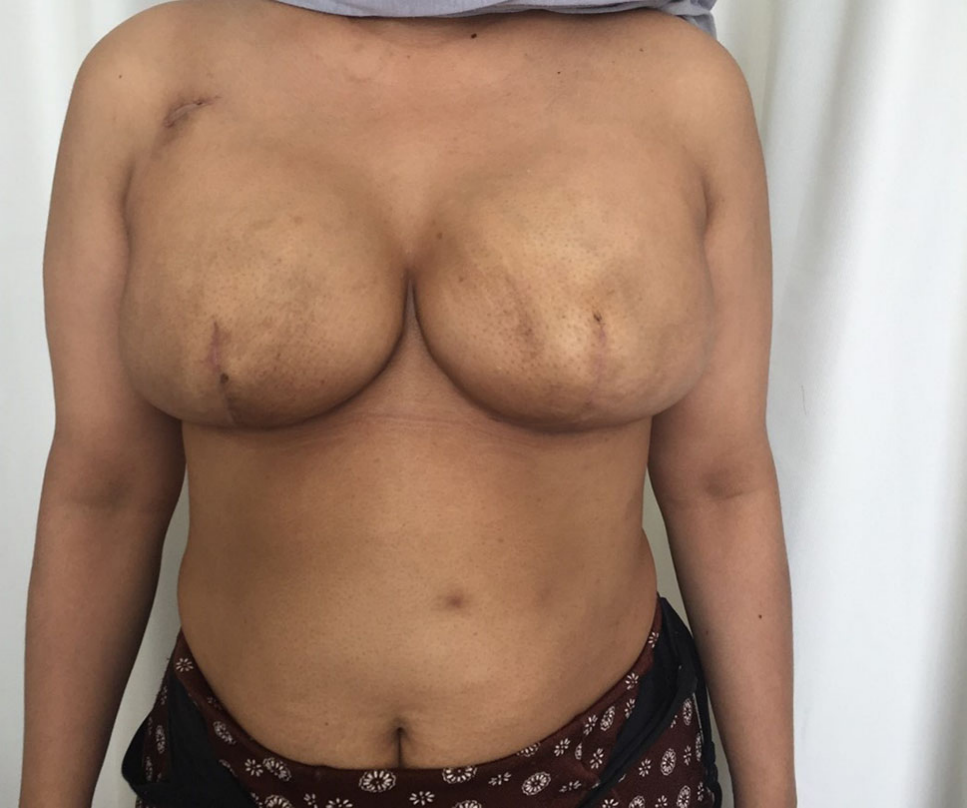
Patient who underwent IMF manipulation and tissue expanders inserted, now 8 months post operation. The photograph shows a crisp and well defined IMF

**Fig. 3:**
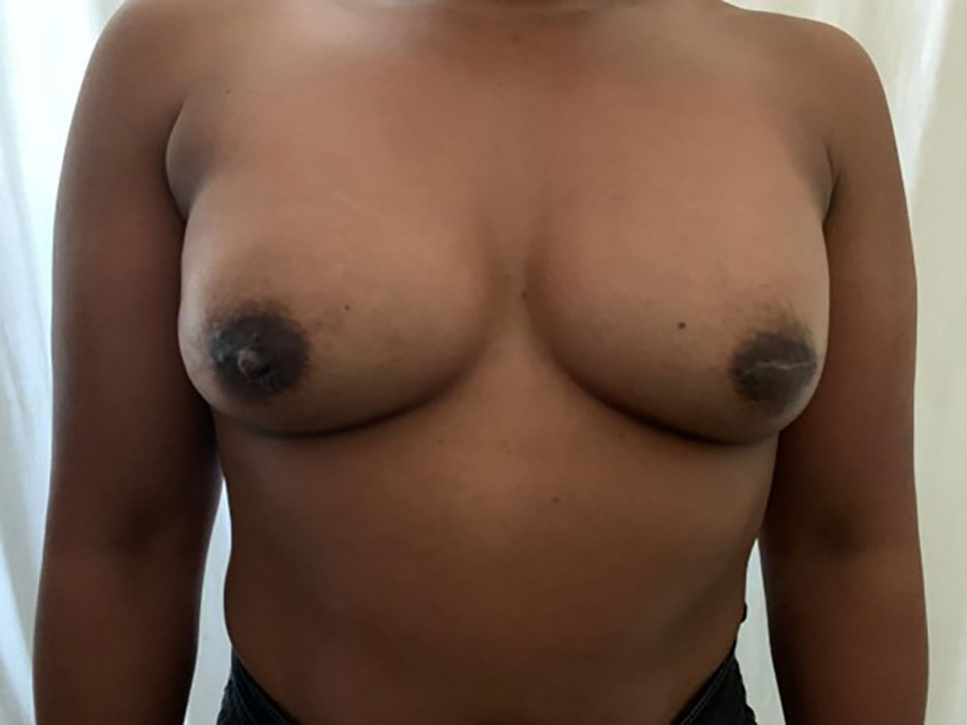
Patient who had bilateral direct to implant, now 6 months post op, showing a crisp, semi-circular IMF. On the left side the nipple was excised during mastectomy

**Fig. 4:**
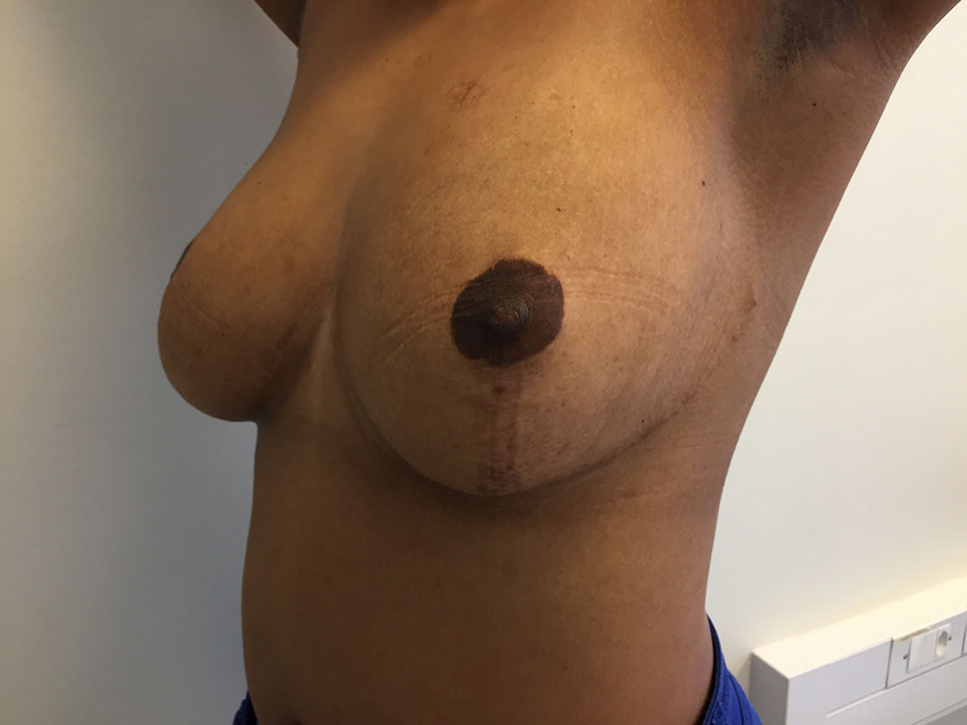
Patient after bilateral breast reduction 38 months post op. Note how the lateral IMF curves superiorly to end at the same level as the medial IMF. IMF mobilized, repositioned, and sutured

In both situations, these sutures result in a crisp, semi-elliptical shape of the IMF (Figs. 2, 3, 4)

## Results

There were 56 patients: 43 having bilateral mastectomy and immediate breast reconstruction, and 13 having bilateral breast reductions. (Table 1).

### Breast Reduction

Thirteen patients, mean age of 36 years (range 21-65 years) underwent breast reduction.

In 10 patients, the lateral IMF was lower than the medial IMF (mean 1.8 cm, range 1–3 cm) and equal in 3 patients. In all cases, a Wise keyhole skin pattern with superomedial pedicle was used.

The mean follow-up was 11 months (range 6–36 months)

### Breast Reconstruction

Forty-three patients who underwent mastectomy and immediate reconstruction, and 30 patients had inferior displacement of the lateral IMF (mean 2.9 cm, range 2–7 cm). The mean age was 42 years (range 30–61 years). The follow-up of these patients was 13 months (range 3–36 months)

## Discussion

The definition and shape of the inframammary fold (IMF) play a crucial role in determining breast aesthetics [1–3]. Although there is uncertainty regarding its histological composition [7–11], it consists of both a subcutaneous component (connecting dermis to SFS) and a deeper component (connecting SFS to the pectoral fascia/ribs). These studies suggest that the IMF exists primarily as a subcutaneous structure, where it is better formed and more clinically evident, whereas the deeper component is more tenuous.

Interestingly, recent anatomical studies have supported the clinical observation that the lateral IMF may have a lower and more lateral insertion point compared to the medial IMF, as shown in Table 1. Takaya et al. [3] reported that the IMF becomes less prominent as it reaches the anterior axillary line. Gaskin et al. [9] observed that the breast tends to project outward and inferiorly with age. Both studies were conducted on cadavers and did not include patients with macromastia.

The morphology of the IMF should be critically assessed in all patients having breast surgery. In some patients blunting of the lateral IMF may even lead to displacement of the NAC from the breast meridian [6]. The loss of the semi-circular curve to the IMF is less apparent when the patient is supine and may explain why this clinical observation has not received more attention.

### Breast Reduction

In macromastia, and particularly in patients with a high BMI, the entire IMF is expanded by the excess adiposity. This leads to a diffuse IMF, with more fat particles between the fibrous structure forming the IMF3. Consequently, this expanded structure loses definition and becomes weakened. Also, the weight of the enlarged breast causes the IMF to descend 5, 6. By surgically compressing the attenuated and expanded IMF using sutures, its definition is restored. The IMF suture technique described in which the suture passes from deep fascia to SFS and then travels in a subcutaneous plane (then returns back to the SFS and then finally to the deep fascia, and tied) actually mimics the anatomical structure of the IMF. Once tied, this technique concertina’s this structure back to its original configuration. In breast reduction, using an inframammary incision is used (inverted T/keyhole, used in all cases in this study) attenuates the IMF. Therefore, the IMF suture is designed to strengthen and support it [12, 13], while also preventing its descend due to the weight of the repositioned breast tissue above it.

In some patients with macromastia, the shape of the IMF is altered. The hemi-elliptical shape, where the medial aspect and lateral aspect of the fold are at the same horizontal level no longer occurs. Particularly, the lateral aspect of the IMF descends, resulting in a flat and somewhat amorphous shape. This is again accentuated in patients with a raised BMI [6]. This was noted in three quarters of patients having a bilateral breast reduction in this study. Therefore, these patients requires repositioning of the displaced IMF into a more superior position. Consequently, in these patients, the IMF needs to be both reinforced and repositioned.

### Breast Reconstruction

A major aesthetic advantages of the skin sparing and nipple sparing mastectomy are preservation of the inframammary fold, which is crucial for breast aesthetics. Regardless of the skin pattern used, a mastectomy attenuates the IMF. This can be corrected by suture reinforcement, which is especially important during reconstruction, whether using implants [13] or autologous [14] methods. The more sutures that are inserted, the stronger the fixation, and from an aesthetic perspective, this restores the crispness and definition of the IMF (Fig. 4).

Breast cancer typically affects middle aged and older women, and it is in these patients that the ideal configuration of the IMF may be lost. Specifically, there is inferior displacement of the lateral IMF, necessitating both repositioning the IMF in a more superior position and then reinforcing it. Displacement of the lateral segment of the IMF was noted to occur in 70% of patients having breast reconstruction in this study.

Disadvantages of IMF fixation include the fact that these sutures cause more post-operative pain, but this resolves with analgesia. In patients with a high BMI, repositioning the displaced lateral IMF may exacerbate the lateral dog ear when using a keyhole design. Liposuction of the axilla prevents this from occurring.

There are some shortcomings to this article. Short-term (median follow-up 1 year) results have been satisfactory. However, the long-term outcome has yet to be established. This is hindered by the lack of reliable methods for plotting changes in IMF configuration. Additionally, various factors, including weight fluctuation, etc. can impact long-term outcomes in the breast. Only one patient in this study, who had had a breast reduction, developed pseudo ptosis. Moreover, if the IMF sutures are not inserted correctly, scalloping of the IMF may occur.

This study suggests that assessing the configuration of the IMF is important before undertaking breast surgery. Where the IMF is attenuated but its shape is retained, the IMF should be reinforced. When the lateral IMF is inferiorly displaced, resulting in a flattened/shallow curve with loss of definition, it should be mobilized, repositioned then sutured into a more aesthetically pleasing position.

### Supplementary Information

Below is the link to the electronic supplementary material.

Video 1 Patient X due to undergo bilateral nipple sparing mastectomy. Note that the lateral IMF is displaced inferiorly and is below/inferior to the medial IMF (i.e. if a line was drawn across the breast at the level of the medial IMF, this line would pass above the superior extent of the lateral IMF (MOV 11905 KB)

Video 2 Video of patient X above, who underwent nipple sparing mastectomy using keyhole pattern with inferior deepithelialised flap. Exaggerated traction on the IMF sutures shows the lateral IMF moving superiorly. These sutures are then tied with the lateral IMF in a more cranial/superior position. (MOV 37677 KB)

Video 3 Intraop video of patient X, after IMF mobilisation, repositioning, and suture reinforcement. Note a better configuration of the IMF, i.e. more semi-circular in shape. (MOV 18832 KB)
